# 282. Resistance to caspase-1 dependent pyroptosis of hypervirulent *K. pneumoniae*

**DOI:** 10.1093/ofid/ofad500.354

**Published:** 2023-11-27

**Authors:** Hyun ah Kim, Miri Hyun, Jiyeon Lee, Jin Kyung Kim, wonki baek

**Affiliations:** Keimyung University Dongsan hospital, Daegu, Taegu-jikhalsi, Republic of Korea; Keimyung University School of Medicine, Daegu, Taegu-jikhalsi, Republic of Korea; Keimyung University Dongsan hospital, Daegu, Taegu-jikhalsi, Republic of Korea; Keimyung University School of Medicine, Daegu, Taegu-jikhalsi, Republic of Korea; Keimyung University School of Medicine, Daegu, Taegu-jikhalsi, Republic of Korea

## Abstract

**Background:**

Hypervirulent *K. pneumoniae(*Hv Kp*)* has emerged as a clinically significant global pathogen in the last decade. However, the host immune responses of the macrophages during hvKp infection are largely unknown. In the present study, we aimed to compare the cytotoxic effects of hvKp and classical *K. pneumoniae* (cKp) in murine macrophages.

**Methods:**

Bacterial strains are obtained from clinical samples who infected by *K. pneumoniae*. The *rmpA* and *iutA* positive strains with mucoid phenotype were defined as hypervirulent *K. pneumoniae*. Raw 264.7 cell and BMDM(bone marrow derived macrophage) cell were used for experiments. Wild-type C57BL/6 female mice and *Casp1*^-/-^ female mice (age, 7–9 weeks) were used. Cytotoxicity after 4 hours of infection, MTS and LDH release was measured. FACS scan after PI dyed was performed for cell cycle analysis and cytotoxicity each MOI(Multiplicity of infection) concentration. Western blot analysis of caspase-1 and caspase-1 inhibitor were used. IL-1β was tested with ELISA.

**Results:**

We found that the activation of caspase-1 (Casp1)-dependent pyroptosis was higher in cKp-infected macrophages compared with that in hvKp-infected macrophages. In caspase-1 deficiency macrophages, pyroptosis diminished during infection. Both hvKp and cKp strains led to nucleotide-binding and oligomerization domain-like receptor protein 3 (NLRP3) inflammasome formation and lysosomal cathepsin B activation, thus resulting in pyroptosis. Compared with the cKp strain, the hvKp strain inhibited these phenomena in murine macrophages.

**Figure 1.**
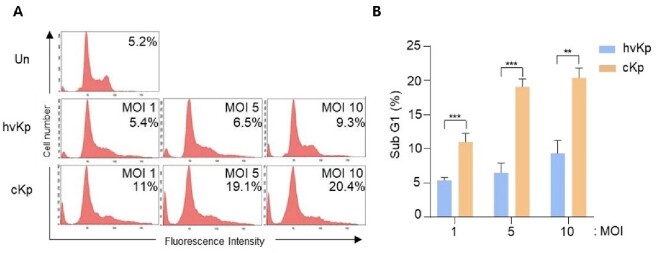
HvKp and cKp strains induce pyroptotic cell death in the RAW264.7 murine macrophage cell line.

**Figure 2.**
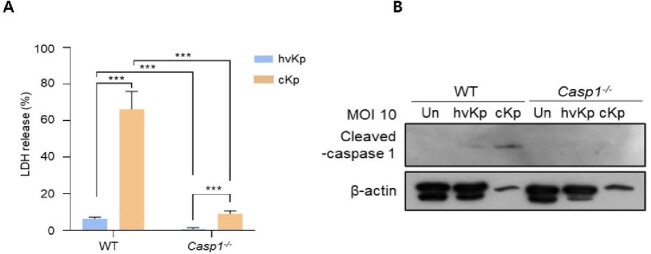
BMDMs were differentiated from WT and Casp1-/- mice and infected with the hvKp or cKp strains.

**Conclusion:**

In this study, compared to classical strains, it was confirmed that cells infected with hv Kp survived without apoptosis. HvKp infection resulted in different levels of pyroptosis via the activation of cathepsin B-NLRP3-caspase-1 in murine macrophage. Therefore, the manipulation of pyroptotic cell death is a potential target for host response during hvKp infection in macrophages.

**Disclosures:**

**All Authors**: No reported disclosures

